# Striatal Dopamine Signals and Reward Learning

**DOI:** 10.1093/function/zqad056

**Published:** 2023-10-03

**Authors:** Pol Bech, Sylvain Crochet, Robin Dard, Parviz Ghaderi, Yanqi Liu, Meriam Malekzadeh, Carl C H Petersen, Mauro Pulin, Anthony Renard, Christos Sourmpis

**Affiliations:** Laboratory of Sensory Processing, Brain Mind Institute, Faculty of Life Sciences, Ecole Polytechnique Fédérale de Lausanne (EPFL), Lausanne CH-1015, Switzerland; Laboratory of Sensory Processing, Brain Mind Institute, Faculty of Life Sciences, Ecole Polytechnique Fédérale de Lausanne (EPFL), Lausanne CH-1015, Switzerland; Laboratory of Sensory Processing, Brain Mind Institute, Faculty of Life Sciences, Ecole Polytechnique Fédérale de Lausanne (EPFL), Lausanne CH-1015, Switzerland; Laboratory of Sensory Processing, Brain Mind Institute, Faculty of Life Sciences, Ecole Polytechnique Fédérale de Lausanne (EPFL), Lausanne CH-1015, Switzerland; Laboratory of Sensory Processing, Brain Mind Institute, Faculty of Life Sciences, Ecole Polytechnique Fédérale de Lausanne (EPFL), Lausanne CH-1015, Switzerland; Laboratory of Sensory Processing, Brain Mind Institute, Faculty of Life Sciences, Ecole Polytechnique Fédérale de Lausanne (EPFL), Lausanne CH-1015, Switzerland; Laboratory of Sensory Processing, Brain Mind Institute, Faculty of Life Sciences, Ecole Polytechnique Fédérale de Lausanne (EPFL), Lausanne CH-1015, Switzerland; Laboratory of Sensory Processing, Brain Mind Institute, Faculty of Life Sciences, Ecole Polytechnique Fédérale de Lausanne (EPFL), Lausanne CH-1015, Switzerland; Laboratory of Sensory Processing, Brain Mind Institute, Faculty of Life Sciences, Ecole Polytechnique Fédérale de Lausanne (EPFL), Lausanne CH-1015, Switzerland; Laboratory of Sensory Processing, Brain Mind Institute, Faculty of Life Sciences, Ecole Polytechnique Fédérale de Lausanne (EPFL), Lausanne CH-1015, Switzerland

**Keywords:** Reward-based learning, dopamine, neuronal circuits, sensory processing, whisker sensory perception, motor control, licking, goal-directed behavior, striatum, synaptic plasticity

## Abstract

We are constantly bombarded by sensory information and constantly making decisions on how to act. In order to optimally adapt behavior, we must judge which sequences of sensory inputs and actions lead to successful outcomes in specific circumstances. Neuronal circuits of the basal ganglia have been strongly implicated in action selection, as well as the learning and execution of goal-directed behaviors, with accumulating evidence supporting the hypothesis that midbrain dopamine neurons might encode a reward signal useful for learning. Here, we review evidence suggesting that midbrain dopaminergic neurons signal reward prediction error, driving synaptic plasticity in the striatum underlying learning. We focus on phasic increases in action potential firing of midbrain dopamine neurons in response to unexpected rewards. These dopamine neurons prominently innervate the dorsal and ventral striatum. In the striatum, the released dopamine binds to dopamine receptors, where it regulates the plasticity of glutamatergic synapses. The increase of striatal dopamine accompanying an unexpected reward activates dopamine type 1 receptors (D1Rs) initiating a signaling cascade that promotes long-term potentiation of recently active glutamatergic input onto striatonigral neurons. Sensorimotor-evoked glutamatergic input, which is active immediately before reward delivery will thus be strengthened onto neurons in the striatum expressing D1Rs. In turn, these neurons cause disinhibition of brainstem motor centers and disinhibition of the motor thalamus, thus promoting motor output to reinforce rewarded stimulus-action outcomes. Although many details of the hypothesis need further investigation, altogether, it seems likely that dopamine signals in the striatum might underlie important aspects of goal-directed reward-based learning.

## Graphical Abstract

**Graphical Abstract:**
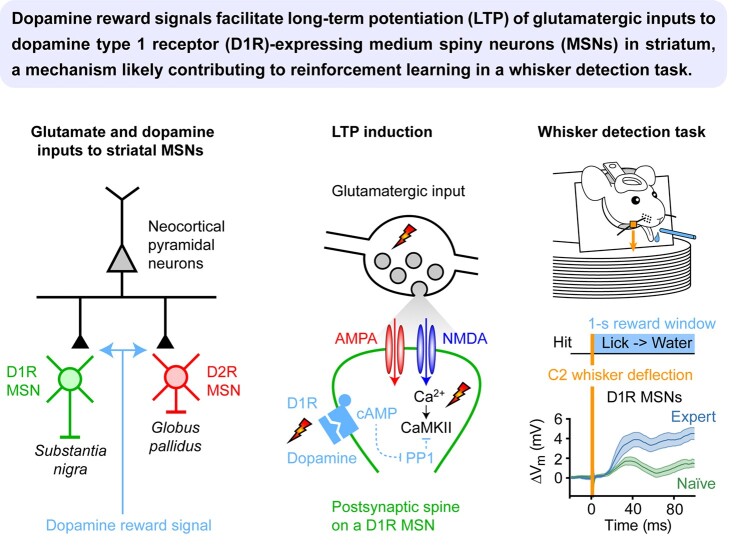

## Reward-based Reinforcement Learning

**Figure 1.**
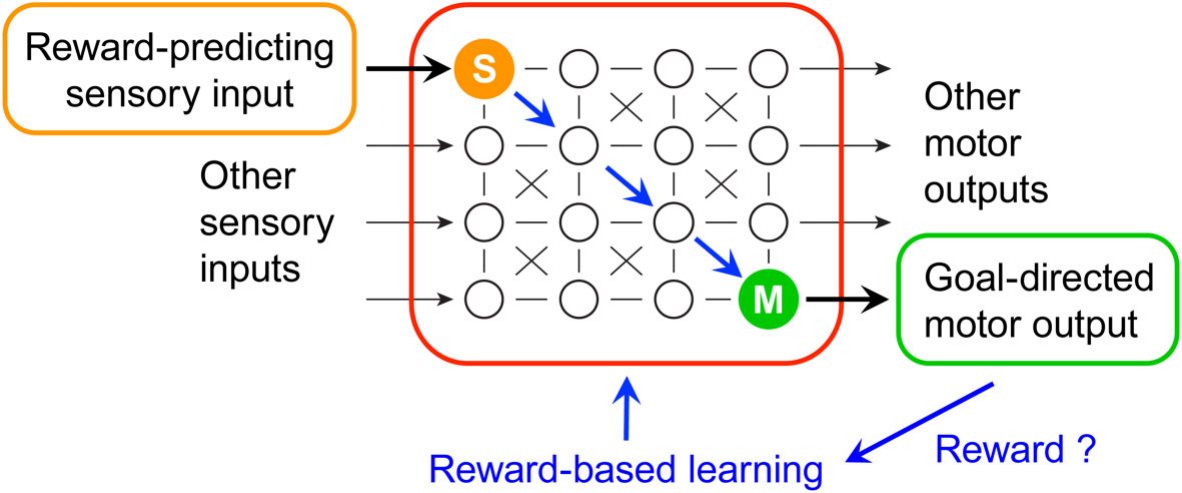
Learning sensory-to-motor transformations from rewards. Animal behavior is determined by incoming sensory information, innate neuronal circuits, short- and long-term memories, and internal states. In part, actions are tuned to maximize reward. Animals can learn to obtain rewards by responding with appropriate goal-directed motor output to relevant reward-predicting sensory input in specific contexts through trial-and-error reward-based learning. Reward signals (blue) are thought to drive synaptic plasticity in neuronal circuits, such that relevant sensory signals in sensory neurons (S, orange) drive appropriate motor output controlled by motor neurons (M, green) in order to receive reward.

The brain constantly receives sensory input while governing motor output (Figure 1). Sensory input to the brain provides both external and internal information. Information about internal states, such as thirst, provides motivation for goal-directed behavior. External cues may help animals learn when, where, and what to do in order to obtain a reward, such as how to obtain water if thirsty. If a specific action is rewarded in a given sensory context, then it might be important for an animal to learn and reinforce such a stimulus-action-reward coupling. For such learning to occur, neuronal circuits in the brain must change so that the relevant sensory neurons signal to the correct motor neurons in order to execute the appropriate goal-directed sensory-to-motor transformations. Such reward-based sensorimotor learning is not a trivial process for neuronal networks because: (i) reward is necessarily delayed relative to action initiation and sensory processing; (ii) animals constantly receive multimodal sensory information and are constantly in motion; and (iii) primary rewards are typically sparse in natural conditions. Thus, assuming that animals are trying to maximize their future-obtained reward, the brain should learn and reinforce the sequence of sensorimotor events yielding the highest reward probability.

Experimentally, in trial-based reward learning, subjects are typically presented with sensory information and need to perform a specific action in order to obtain a reward. The rewarded stimulus, the sensory context, and the required action might not be known to the subject in the first place and needs to be discovered through trial-and-error learning. Thus, there must be neuronal signals in the brain that encode the value of each trial outcome (rewarding, aversive, or neutral). If the outcome is unexpectedly positive/negative, then brain circuits should be modified to reinforce/reduce the link between these successful/unsuccessful sequences of sensory events and motor commands.

Interesting insights into the types of signals that could drive such bidirectional modulation come from the conversation between the fields of neuroscience and reinforcement learning. In the latter, rewards are used to learn associations between actions and outcomes and to use that information to maximize total future rewards. While many reinforcement learning methods are successful at learning such associations, temporal difference (TD) learning is of particular interest for neuroscience^1^, ^2^ due to its capacity to assign credit to sequences of events leading to reward and its sensitivity toward the temporal relationships between stimulus and outcomes.

In early models of reward-based learning, the primary focus was on establishing associations between conditioned stimuli and actions leading to reward. These models, initially formalized by Bush and Mosteller,^3^, ^4^ proposed that classical associations strengthen through the iterative computation of an error term that captures the difference between the reward an animal received at the current trial and what was experienced following previous presentations of the stimulus


\begin{eqnarray*}
{A}_{i + 1} = \ {A}_i + \ \alpha \left( {{r}_{i + 1} - \ {A}_i} \right) ,
\end{eqnarray*}

where ${A}_i$ represents the strength of the association between the conditioned stimulus and the action at trial $i$; ${r}_i$ represents the obtained reward at trial $i$; and $\alpha \in [ {0,1} ]$ is the learning rate.

This formulation was famously employed in the Rescorla–Wagner model of Pavlovian conditioning,^5^ but was limited in scope as it boils down to computing a weighted average of past rewards. As such, it is unable to account for a wide array of phenomena commonly observed in psychology, where various events occurring within a trial convey distinct information about reward availability. In particular, it fails to learn second-order conditioning, that is, learning that if a primary cue predicts reward and the occurrence of a secondary cue predicts the primary cue, then the secondary cue becomes predictive of reward and the appearance of the primary cue does not convey new information. A fundamental shift in understanding reward-based learning occurred with the introduction of TD learning by Sutton and Barto to address these problems.^1^, ^2^ Temporal difference learning departs from Rescorla and Wagner’s approach in two ways. First, it breaks down the trial structure into a series of $n\ $ discrete time steps (referred to as states), $s = 1 \le i \le N,$ enabling learning not only at the end of a trial after reward delivery, but at each moment. Second, instead of focusing on learning the value of past events, TD learning formulates the problem as predicting the value of future ones. At each time point $t$, where one of the states $s\ $ is visited, it makes a prediction of the expected future reward, referred to as the *value* of that state. After learning occurs, these predictions will converge to the expected sum of the currently expected reward and the discounted reward of all future time points


\begin{eqnarray*}
{V}_t &=& E\left({{r}_t + {\rm \gamma}{r}_{t + 1} + {\gamma }^2{r}_{t + 2} + \ldots } \right)\nonumber\\
&&\quad {V}_t = E({r}_t) + \gamma {V}_{t + 1},
\end{eqnarray*}

where ${V}_t$ is the value at $t$; ${r}_t$ is the obtained reward at $t$, and $\gamma \in [ {0,1} ]$ is the discount rate ensuring that the sum is finite by discounting rewards coming far in the future over nearby ones and $E$ is the mathematical expectation.

The core concept in learning these values (${V}_t$) lies in the discrepancy between the obtained and the predicted reward. The difference is termed *reward prediction error* (RPE) and serves as the teaching signal used to adjust ${V}_t$


\begin{eqnarray*}
{\delta }_t = {r}_t + \gamma {V}_{t + 1} - {V}_t ,
\end{eqnarray*}

where ${V}_t$ is the value of the state visited at time $t$ and ${r}_t$ is the obtained reward at that time. When an unexpected reward is obtained, it creates a discrepancy between the reward currently expected at that state and the reward actually obtained. This positive difference leads to what is commonly referred to as a *positive* RPE. If a punishment is delivered or the obtained reward is lower than expected, then a *negative* RPE is generated. Both positive and negative RPE are used in the adjustment process of ${V}_t$ according to the following update rule, with $\alpha $ indicating the learning rate:


\begin{eqnarray*}
{V}_t \leftarrow {V}_t + \alpha {\delta }_t .
\end{eqnarray*}

To learn that the presentation of a cue predicts a reward, the system adjusts the value of the cue so that an RPE is generated at the cue presentation. This is achieved by treating cue presentation and reward delivery as two distinct time steps and, critically, by estimating at the time of stimulus presentation not only the immediate reward (which never comes at that time), but also the reward expected at the later step of delivery. In other words, this process allows the generation of RPEs not only when an unexpected reward is obtained but also when unexpected information signaling a reward is received, effectively propagating backwards in time the RPE of the rewarded state toward the predictive cue. As reviewed below, the functional role of RPEs as defined in TD learning closely aligns with the firing rate of dopaminergic neurons in the midbrain.^6–9^

It is crucial to note that while rewards can be delivered frequently in laboratory settings, typically in the form of food or water to hungry or thirsty animals, in nature, physical rewards can be very sparse, which makes learning difficult for animals as well as for artificial agents. Recently, there have therefore been efforts to expand the definition of reward and introduce other concepts that could serve as additional learning factors such as curiosity,^10^ surprise,^11^ or novelty.^12^

## Midbrain Dopamine Neurons Signal Reward Prediction Errors

**Figure 2.**
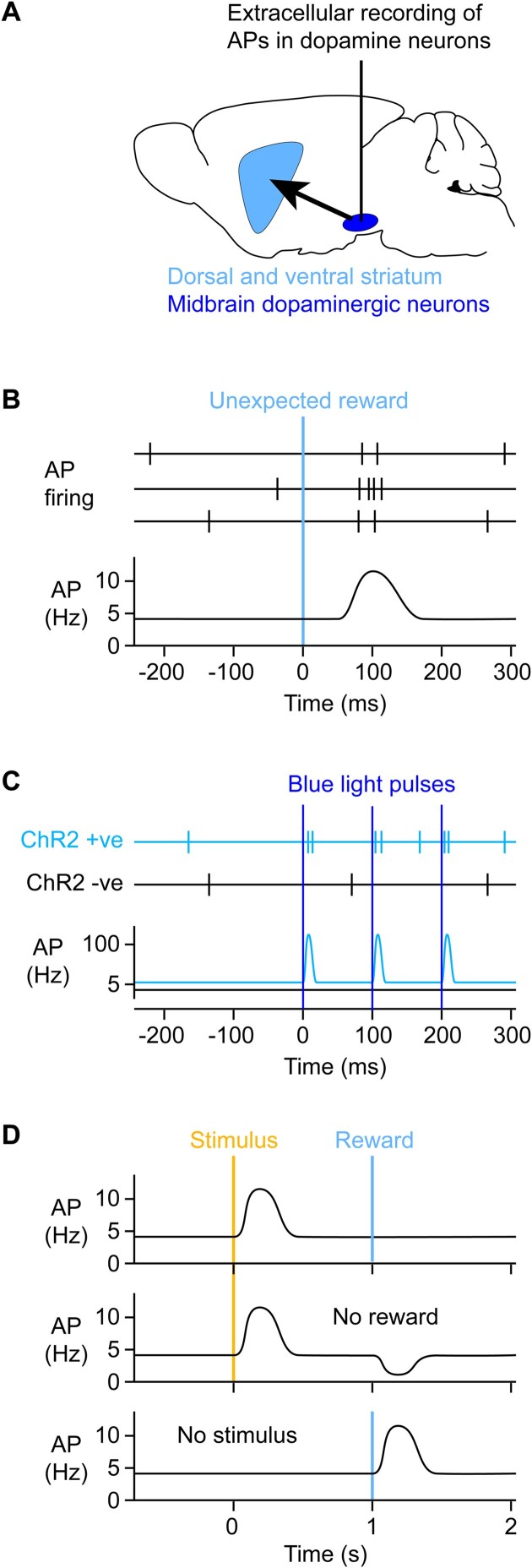
Optogenetically identified dopamine neurons in the midbrain transiently increase firing in response to unexpected rewards. (A) In order to study their activity, extracellular electrophysiological recordings can be targeted to the midbrain dopaminergic neurons (dark blue) located in the substantia nigra pars compacta and the VTA, which respectively prominently innervate the dorsal striatum and ventral striatum, also known as the nucleus accumbens (light blue). (B) The work of Wolfram Schultz and colleagues revealed that delivery of an unexpected reward transiently increases AP firing in putative midbrain dopamine neurons of monkeys.^6^ (C) Opto-tagging can be used to record AP firing of genetically identified classes of neurons. For example, the light-gated ion channel ChR2 can be expressed specifically in dopaminergic neurons of the midbrain through mouse genetics and viral transfection. Blue light flashes specifically drive AP firing in ChR2-expressing neurons, which can be recorded by an optrode, a device consisting of an optical fiber coupled to an extracellular recording electrode. Blue light delivery can evoke precisely timed AP firing in subsets of neurons expressing ChR2. The work of Naoshige Uchida and colleagues studied opto-tagged dopaminergic neurons and found that such genetically defined dopaminergic neurons transiently increased AP firing in response to rewards in mice,^8^ as shown in panel B and similar to the previous work in monkeys.^6^ (D) Substantial evidence supports the hypothesis that dopamine neurons do not only respond to unexpected rewards, but more precisely they encode RPEs. Animals can learn that specific sensory stimuli reliably predict future rewards. After learning, the reward-predicting sensory stimulus evokes a rapid transient increase in dopamine neuron AP firing, but there is no dopamine signal upon reward delivery because it is now entirely expected (top). However, if reward is omitted, then there is a drop in dopamine firing rates because the outcome was worse than expected (negative RPE) (middle). On the other hand, if the reward-predicting sensory stimulus is omitted, then reward delivery is unexpected and is again accompanied by increased dopamine neuron firing (below).

The most prominently described reward signal in the mammalian brain comes from midbrain dopaminergic neurons located in the substantia nigra pars compacta (SNc) and the adjacently lying ventral tegmental area (VTA) (Figure 2A). These midbrain dopaminergic neurons strongly project to the dorsal and ventral striatum, with SNc dopamine neurons projecting to the dorsal striatum and VTA dopamine neurons mainly projecting to the ventral striatum, also termed the nucleus accumbens. The activity of SNc/VTA neurons can be observed through precisely targeted extracellular electrophysiological recordings of neuronal action potential (AP) firing during animal behavior.^13–15^ As first described in monkeys by Schultz et al.^6^, AP firing rates of some SNc/VTA neurons rapidly and transiently increase in response to unexpected rewards (Figure 2B) and more generally were found to signal RPE,^6^ which had been identified as an important learning signal associated with octopamine in the honey bee.^16^, ^17^ There are different types of neurons in the SNc/VTA that can be distinguished by distinct molecular, structural, and functional features. An important advance supporting the dopamine reward coding hypothesis came from the work of Naoshige Uchida’s laboratory through electrophysiological recordings of optogenetically identified dopaminergic neurons.^8^ In this method, the light-gated cation channel channelrhodopsin-2 (ChR2) is specifically expressed in dopaminergic neurons by injecting Cre-dependent adeno-associated virus (AAV) into the SNc/VTA of dopamine reuptake transporter (DAT)-Cre mice (Figure 2C). These mice express Cre-recombinase in cells that express the plasma membrane DAT, a key signature of dopaminergic neurons. Blue light flashes delivered to the midbrain through fiber optics can then evoke activity by directly depolarizing the ChR2-expressing dopamine neurons. Optogenetic stimulation evoked precisely timed AP firing in opto-tagged SNc/VTA neurons in DAT-ChR2 mice, thus genetically defining them as dopaminergic. These optogenetically defined dopaminergic neurons had AP firing patterns consistent with transiently increased activity in response to unexpected rewards and RPE (Figure 2B).^8^

As discussed in the previous section, signals representing unexpected reward and RPE are useful for reinforcement learning, and dopamine neurons could therefore causally serve to deliver such learning signals. To test this hypothesis, it is essential to directly manipulate the activity of dopamine neurons. Current experimental data largely supports the notion that artificially induced transient increases in dopamine indeed act to positively reinforce behavior. By expressing ChR2 in dopamine neurons, dopamine concentrations can be increased through blue light stimulation, similar to the opto-tagging experiments described above. Optogenetic stimulation of dopamine neurons was found to induce place preference, such that a mouse would spend more time in the region of the test chamber in which the dopamine neurons were driven to fire at high frequency.^18^ Mice were also found to reinforce nose-poking when this triggered optogenetic stimulation of dopaminergic neurons.^19^ In an operant training behavior, mice would also learn to press a lever in order to self-stimulate dopamine neurons optogenetically, with some mice persevering with dopamine self-stimulation even when this was paired with footshock.^20^ In head-restrained mice carrying out a visual discrimination task in order to receive reward, stimulation of dopamine neurons appeared to enhance learning speed and prolonged session duration.^21^ Optogenetic stimulation of dopamine neurons was also found to evoke orofacial movements that share similarities to those time-locked to reward-predicting cues in animals trained in stimulus-reward association tasks.^22^ Finally, specific spontaneous movements occur more frequently in a behavioral session if they were previously paired with optogenetic stimulation of dopaminergic axons in the dorsolateral striatum (DLS).^23^ A large body of evidence therefore supports the notion that increases in dopamine are rewarding and act as positive reinforcers. Conversely, reduced firing of dopamine neurons, either by stimulating GABAergic inputs to the dopamine neurons^24^ or direct optogenetic inhibition of dopamine neurons^25^ can cause aversion, acting as a negative reinforcer.

Interestingly, dopamine not only signals unexpected primary rewards, such as water for a thirsty animal, but, through learning, dopamine signals develop in response to cues that predict future reward (Figure 2D).^6^ If a sensory cue is repeatedly presented to a subject in a manner that anticipates reward delivery, then dopamine neurons will shift their responses to the earliest time point predictive of upcoming rewards. This computation is useful for optimally learning rewarded sensorimotor sequences.^6^, ^7^, ^26^, ^27^ This shift in their response profile upon learning strongly reflects what one would expect from an RPE signal, according to the TD learning model. As mentioned in the previous section, in TD learning, RPE is obtained from the difference between the observed and predicted values of the current state. When we consider this computation performed moment-by-moment in a time-continuous setting, the result is that TD-RPE approximates the derivative of the value function. As unexpected rewards are presented, the sudden increase of value associated with that state generates a positive RPE, which propagates to the predictive cue over learning. At that stage, dopamine neurons will respond to the increase in rate of change of the value function associated with the presentation of the cue that is only resolved at the time of reward presentation.

Indeed, as a reward becomes expected, the response of dopamine neurons to reward scales as a function of the difference between the obtained and the expected reward, that is, dopamine neurons increase their response to reward when the reward is bigger than expected and decrease when the reward is smaller than expected, even going below baseline firing rates in case of reward omission.^6^, ^8^, ^9^, ^28^, ^29^ If the sensory cue predicts upcoming reward with complete certainty, then the reward delivery itself becomes entirely expected, and thus, the dopamine signal at the reward delivery time decreases.^7^, ^26^ This is well in accordance with the RPE signaling hypothesis and implies that with experience, the subject builds an internal model that associates an expected value (reward probability and size) to a given sensorimotor sequence in a specific context. Whereas in monkey studies dopamine signals for fully predicted reward completely disappear, this has typically not been observed in mouse experiments, perhaps because mice have noisier time estimation abilities or may have been less extensively trained and thus remain more uncertain about reward expectations.^8^ Indeed, even in monkeys, if the sensory cue is only partially predictive, then the partially predicted reward evokes a dopamine signal, although smaller compared to that evoked by delivery of the same reward at an unexpected time, and the sensory cue evokes a reduced dopamine signal compared to fully reward-predicting cues.^7^, ^26^

What drives RPE signals in midbrain dopamine neurons? One major input that dopamine neurons receive comes from nearby inhibitory GABAergic neurons. Dopaminergic neurons form approximately 60% of the neuronal population in VTA, and the rest are mostly GABAergic inhibitory neurons, with a smaller fraction of glutamatergic neurons.^30^ GABAergic neurons in the VTA are more represented in the rostral and medial parts. Electrophysiological recordings from opto-tagged GABAergic neurons located in the vicinity of the VTA revealed that these inhibitory neurons appeared to encode expectation about rewards, but were not strongly affected by reward delivery or omission.^8^ Whereas dopamine neurons in VTA represent the difference between observed and predicted reward, the GABAergic neurons predominantly represent only the predicted reward. Optogenetic manipulations of the VTA GABAergic neurons directly showed that these neurons provided inhibition to the VTA dopamine neurons in a subtractive manner, such that the predicted reward is subtracted from the actual reward in order to compute RPE.^31^

In addition to input from local inhibitory neurons, dopamine neurons receive a multitude of inputs from other brain regions. These can be identified in a brain-wide manner by using monosynaptically restricted mapping of presynaptic neurons labeled by a modified rabies virus.^32^ Applying this method to specifically trace inputs to midbrain dopamine neurons revealed many inputs, including from the cortex, basal ganglia, amygdala, hypothalamus, midbrain reticular formation, periaqueductal gray, superior colliculus, dorsal raphe, and the parabrachial nucleus.^33–35^ Ventral tegmental area dopamine neurons have been shown to receive glutamatergic inputs from the cortex,^36^ brainstem,^37^ midbrain,^38^ basal forebrain,^39^ and dorsal raphe nucleus,^37^ and GABAergic inputs from the lateral hypothalamus,^40^ brainstem,^38^ dorsal raphe nucleus,^35^ and ventral pallidum.^41^ Interesting differences were also observed comparing the inputs to SNc and VTA dopamine neurons.^33^, ^34^ Whereas SNc dopamine neurons receive stronger input from the dorsal striatum, globus pallidus, subthalamic nucleus, substantia nigra pars reticulata (SNr) and sensorimotor neocortex, VTA neurons receive stronger input from ventral striatum, ventral pallidum, lateral habenula (LHb), prefrontal and orbital cortex, hypothalamus, and dorsal raphe. These differences in their inputs likely contribute to their differential activity patterns, as discussed later.

The neuronal circuitry and function of LHb inputs have been studied in some detail. Glutamatergic LHb neurons were found to be excited by reward omission, while reward-related stimuli inhibit their activity.^42^ In doing so, the LHb governs the activity of the downstream dopaminergic reward system, mainly through a disynaptic pathway relayed via midbrain GABAergic neurons of the rostromedial tegmental nucleus (RMTg), also known as the tail of the VTA.^43^ The RMTg neurons show phasic activation in response to aversive stimuli like footshocks, shock-predictive cues, food deprivation, or reward omission, whereas they are inhibited after rewards or reward prediction.^44^ As such, inhibition of LHb activity during reward stimuli would reduce the excitation of GABAergic RMTg neurons, leading to disinhibition of dopaminergic VTA neurons. This neuronal circuitry may play a key role in reward learning since habenula lesions were found to impair midbrain dopamine neurons from encoding reward omission in a reward-conditioning task.^45^ In support of these observations, it was recently reported that reward-predictive cues drive LHb inhibition mediated by fast GABAergic neurotransmission, which increases as reward-anticipatory behavior emerges.^46^ Although there is growing evidence that LHb participates in reward-based learning through a LHb-RMTg-VTA circuit,^46^, ^47^ the upstream synaptic inputs onto LHb neurons active during these behaviors currently remain less clear.

As an alternative to the extracellular electrophysiological measurement of the somatic AP firing of dopamine neurons, it is also possible to image the activity of dopamine axons,^48^ or to directly image dopamine release,^49^, ^50^ revealing interesting spatiotemporal dynamics of dopaminergic signaling in the striatum. These methods have largely replaced previous efforts to measure dopamine through microdialysis or voltammetry. Genetically encoded fluorescent calcium indicators, such as GCaMPs,^51^, ^52^ can be expressed in midbrain dopamine neurons through combining mouse genetics and viral vectors, and the activity of individual axons can be imaged using two-photon microscopy together with invasive cranial windows or bulk signals can be measured using fiber photometry.^48^ Recently, it has also become possible to image dopamine release more directly through the development of genetically encoded fluorescent proteins sensitive to dopamine, such as dLight^49^ and GRAB-DA.^50^ Both of these dopamine sensors were engineered to couple a native dopamine receptor to a circularly permuted GFP, rendering fluorescence upon dopamine binding to the receptor. On the whole, the imaging data are consistent with a transient increase in dopamine in the ventral striatum (nucleus accumbens) following an unexpected reward and more generally signaling RPE,^48^, ^49^, ^53^ in good agreement with the electrophysiological measurements. However, other parts of the striatum seem to receive different dopamine signals. Indeed, movement, choice, and motor-related signals may dominate dopamine signaling in the DLS, with a smaller contribution of RPE signals.^48^, ^53–56^ In the striatum, there appears to be a gradient between the ventral and the dorsal parts, with dopamine more prominently signaling movement in the dorsal striatum and reward in the ventral striatum,^48^, ^53^, ^55^ although important reward signals have also been reported in the dorsal striatum.^57^ Indeed, optogenetic stimulation of SNc dopamine neurons, which primarily innervate the dorsal striatum, enhanced movement initiation, whereas optogenetic inhibition of these neurons reduced the probability of movement initiation.^55^ Wave-like propagation of dopamine signals has also been reported through imaging across the dorsal striatum with the directionality changing depending upon task variables.^58^ Additionally, ramping dopamine concentration has also been reported to signal proximity to distant rewards,^59^ which may help enhance motivation and vigor, but these ramping signals can likely also be accounted for by careful consideration of RPE models.^60–62^ Other studies suggest that dopamine neurons in the SNc/VTA may also increase activity in response to novel sensory stimuli, and the increase in dopamine release following a novel stimulus may play an important role in the learning of the association between that stimulus and the reward delivery when the stimulus predicts the reward.^63^ More recently, the tail of the striatum (ie, the most caudal part) has been identified as another region receiving distinct dopaminergic signals. The tail striatum receives dopamine innervation from the most lateral part of the substantia nigra. Rather than encoding reward or movement, the tail striatum dopamine signals seem to function as reinforcers for the avoidance of threatening stimuli.^64^ High-intensity unexpected sound stimuli, but not rewards, drove dopamine increases in the tail striatum, unlike the ventral striatum. Optogenetic stimulation of dopamine fibers in the tail striatum drove aversion, whereas optogenetic stimulation of dopamine fibers in the ventral striatum drove positive reinforcement.^64^ It is therefore clear that there are diverse dopamine signals in different parts of the striatum.

Finally, it is important to remember that although dopamine is considered a key signal for reward learning, it is likely to function in a cooperative manner with several other neuromodulatory systems. For example, in the primary visual cortex of rats, acetylcholine has been shown to be necessary for the learning of the expected time of reward predicted by a visual stimulus during reinforcement learning.^65^, ^66^ This finding was supported by in vitro brain slice experiments in which the activation of metabotropic acetylcholine receptors prolonged the duration of spiking in layer 5 pyramidal neurons evoked by electrical stimulation, extending the time window for synaptic plasticity to occur.^65^ Thus, as described in more detail in the next section, metabotropic signaling by some neuromodulators seems to share common features of promoting synaptic plasticity and learning.

## Dopaminergic Modulation of Synaptic Plasticity in the Striatum

**Figure 3.**
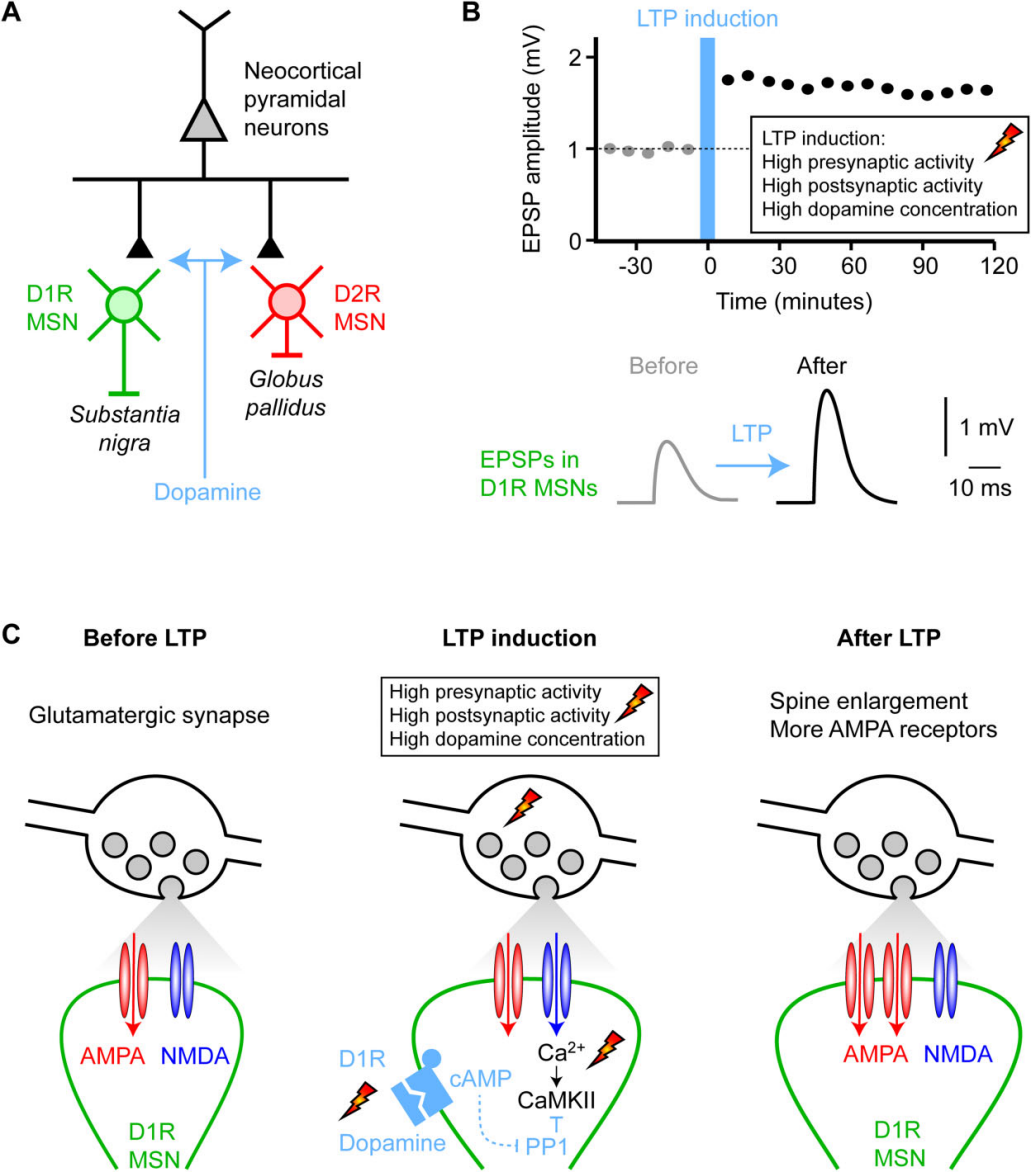
Dopamine modulates synaptic plasticity in the striatum. (A) The midbrain dopaminergic neurons prominently innervate the striatum, which is dominated by two types of GABAergic MSNs expressing different dopamine receptors and projecting to different downstream brain areas. Striatonigral MSNs express D1Rs (green) and project to the SNr. Striatopallidal MSNs express dopamine type 2 receptors (D2Rs, red) and project to the external segment of the globus pallidus. These MSNs also receive glutamatergic input from the cortex and thalamus, and it is thought that a major role of dopamine is to control the plasticity of these glutamatergic inputs to the MSNs. (B) The amplitude of excitatory postsynaptic potentials (EPSPs) onto D1R-expressing MSNs can be increased through long-term potentiation (LTP) induced by pairing presynaptic glutamate release and postsynaptic depolarization together with an increase in dopamine. (C) The mechanisms underlying LTP of glutamatergic synapses on the spines of D1R-expressing MSNs have been studied in detail in brain slice experiments by Haruo Kasai and colleagues.^70^ Three time points are schematically indicated: before, during, and after LTP induction. The upper part of the schematic drawings shows a glutamatergic synaptic bouton filled with synaptic vesicles (gray). The lower part shows a dendritic spine (green) of a D1R-expressing MSN with AMPA (red) and NMDA (blue) subtypes of ionotropic glutamate receptors in the postsynaptic density. In the baseline period (left), AP firing of the glutamatergic afferent causes the release of glutamate evoking a small EPSP in the postsynaptic MSN through the opening of AMPA receptors. NMDA receptors are blocked at resting membrane potential by Mg^2+^. During LTP induction (middle), presynaptic glutamate release is paired with postsynaptic depolarization to open NMDA receptors as well as AMPA receptors. NMDA receptor activation allows Ca^2+^ entry into the spine to activate Ca^2+^/calmodulin–dependent protein kinase II (CaMKII), an essential trigger for many forms of LTP. High activity of protein phosphatase 1 (PP1) would normally inactivate CaMKII under baseline conditions preventing LTP induction, but PP1 is inhibited by elevated cAMP signaling driven by dopamine-activated D1Rs. Thus, dopamine can gate the induction of LTP, resulting in an increased number of AMPA receptors in the postsynaptic density of D1R-expressing MSNs (right).

The activity of dopamine neurons, at least in part, appears to serve as a signal that encodes RPE. In order to understand what impact these dopamine signals might have upon the brain, it is obviously important to consider where dopamine is released. The most prominent target of the axons of the midbrain dopaminergic neurons is the striatum, and it is presumably by releasing dopamine in the striatum that the midbrain dopaminergic neurons carry out an important part of their function. Two classes of striatal projection neurons make up the vast majority of neurons in the striatum, and these two classes of inhibitory GABAergic neurons can be distinguished through anatomical and molecular features, including the expression of different dopamine receptors (Figure 3A).^67^ Striatonigral medium spiny neurons (MSNs) projecting from the DLS to the SNr express dopamine type 1 receptors (D1Rs) and define the so-called direct path. Striatopallidal MSNs projecting from DLS to the external segment of the globus pallidus (GPe) express D2Rs and form the basis of the so-called indirect path.

The striatum receives strong glutamatergic input from the cortex and thalamus. Glutamatergic input to MSNs can change strength through the induction of long-term synaptic plasticity (Figure 3B). The pairing of high-frequency presynaptic firing with high-frequency postsynaptic firing in the presence of elevated dopamine can induce LTP at glutamatergic inputs onto D1R-expressing MSNs, but not D2R-expressing MSNs.^68^, ^69^ Following LTP induction, enhanced efficacy of glutamatergic input increases the amplitude of excitatory postsynaptic potentials (EPSPs) in D1R-expressing MSNs, which can last for many hours. Such long-term synaptic plasticity likely contributes to learning.

The molecular signaling pathways engaged by LTP induction on D1R-expressing MSNs have been investigated in detail by Haruo Kasai and others.^70^, ^71^ (Figure 3C). At baseline, that is, before LTP induction, glutamatergic inputs from the cortex or thalamus would largely activate AMPA receptors located on the spines of the postsynaptic MSNs, giving rise to the electrical signals underlying the EPSP. The voltage-dependent Mg^2+^ block of the NMDA receptors would prevent the calcium-permeable NMDA receptors from conducting current, and, at baseline, there would therefore be little accompanying postsynaptic calcium signaling. During LTP induction, high-frequency presynaptic firing is paired with high-frequency postsynaptic firing. Postsynaptic depolarization releases the voltage-dependent Mg^2+^ block of the NMDA receptors, allowing calcium to enter the dendritic spines of MSNs. Calcium rises are typically considered as the first step in the biochemical cascade underlying the postsynaptic forms of LTP. Elevated spine Ca^2+^ concentrations activate CaMKII, which in turn induces phosphorylation of multiple downstream effectors, culminating in spine enlargement and concomitant insertion of additional AMPA receptors into the postsynaptic membrane, thus giving rise to enhanced EPSPs. However, the activation of CaMKII is countered by a high rate of PP1 activity in MSNs. For D1R-expressing MSNs, increased dopamine concentration activates the D1Rs, which are coupled through the GTP-binding protein G_s_ to stimulate Ca^2+^/calmodulin-dependent adenylyl cyclase 1, in turn increasing intracellular cAMP levels, activating protein kinase A (PKA),^72^ inducing phosphorylation of cAMP-regulated phosphoprotein 32 kD (DARPP-32) and thereby turning PP1 off. A key impact of dopamine in D1R-expressing MSNs therefore seems to be in helping the activation of CaMKII by turning off its inactivation by PP1. Dopamine acting via D1Rs therefore enhances the activation of CaMKII, leading to the induction of LTP at the activated synapses. Interestingly, Haruo Kasai and colleagues^70^ found that the dopamine signals can arrive up to 1 s after the pairing of presynaptic and postsynaptic activity and can still retrogradely enhance LTP through inactivating PP1 to enhance CaMKII activity. This observation is important because rewards are typically delivered after the correct stimulus-response sensorimotor neuronal activity. In order for the dopamine reward signal to contribute to learning through the synaptic plasticity of sensorimotor circuits, it must therefore interact with traces of recent neuronal activity.^73^, ^74^ This is often referred to as the “credit assignment problem” of identifying which synapses should be changed in order to learn and has led to the hypothesis of preferential synaptic plasticity of recently active synapses highlighted by an “eligibility trace,” sharing some similarity to the synaptic tagging hypothesis.^75^ The 1-s window of retrograde enhancement of LTP of recently activated synapses demonstrated in vitro in brain slices could help bridge the time between sensorimotor processing and reward feedback during the learning of simple stimulus-response-reward associations. Altogether, it seems plausible that a delayed dopamine reward signal might trigger plasticity at recently activated synapses, which might have been involved in the sensorimotor activity that gave rise to the reward, thus contributing to reinforcement learning. Similar observations have been made for other neuromodulatory signals, including the effects of norepinephrine and serotonin on the plasticity of cortical glutamatergic synapses^76^ and dopamine affecting synaptic plasticity in the hippocampus,^77^ with experiments showing that neuromodulatory agonists can change the effect of synaptic plasticity induction protocols carried out seconds before the application of the neuromodulatory agonists.

Interestingly, transient decreases in dopamine signals have been reported, especially after reward omissions and more generally in response to negative RPE. Unexpectedly bad outcomes are also important learning signals, and it is therefore also interesting to consider the effects of decreases in striatal dopamine concentration. Dopamine receptor subtypes differ in their affinity for dopamine, with D2Rs having an affinity approximately two orders of magnitude higher than D1Rs. It is thus possible that decreases in dopamine might not be sensed by D1Rs because they may be less activated under basal conditions, and further decreases in dopamine may be outside of the relevant dose-response range of receptor modulation. On the other hand, it may be that D2Rs are normally highly occupied with dopamine even during basal conditions because of their higher affinity. A reduction in dopamine concentration might then lead to a decreased activation of D2Rs. Haruo Kasai and colleagues investigated how such dopamine decreases might affect D2R-related signaling and learning in mice.^71^ D2R activation stimulates G_i/o_ subtypes of G proteins, which inhibit cAMP production and suppress PKA. Whereas increases in dopamine do not appear to drive reductions in PKA activity, decreases in dopamine do evoke increases in PKA activity in D2R-expressing MSNs.^72^ Such dopamine dips appear to be important for mice to carry out a task in which they learn to discriminate between reward-predicting and distractive auditory tones.^71^ The absence of reward in response to the presentation of distractor tones resulted in a reduction in dopamine in the ventral striatum during discrimination learning. The dopamine dip enhanced LTP of glutamatergic inputs onto D2R-expressing MSNs via increased PKA activity, provided concomitant NMDAR-mediated activation of CaMKII and co-activation of adenosine A2A receptors.^71^ Reward omission causing transient reductions in striatal dopamine (perhaps, at least in part, mediated via LHb neurons) might therefore contribute to synaptic plasticity and learning through D2R-expressing MSNs.

It is important to note that dopamine likely does more than modulate the induction of synaptic plasticity in the striatum. Dopamine receptors in MSNs regulate various ionic conductances, including voltage-gated Na^+^, K^+^, and Ca^2+^ channels,^78^, ^79^ with a recent study suggesting that D1R activation increases excitability of striatonigral MSNs largely through voltage- and Ca^2+^-dependent K^+^ channels.^80^ Dopamine receptors are also prominently expressed on presynaptic terminals and other cell classes in the striatum, including the D2Rs on cholinergic interneurons^81^ and astrocytic glial cells.^82^ Finally, dopamine receptors are also found in other brain regions, including frontal cortex, which also receives dopaminergic innervation from VTA neurons. The overall functional role of dopamine signals is therefore likely to be complex.

## Dopamine Signals May Contribute to Reward-Based Learning

**Figure 4.**
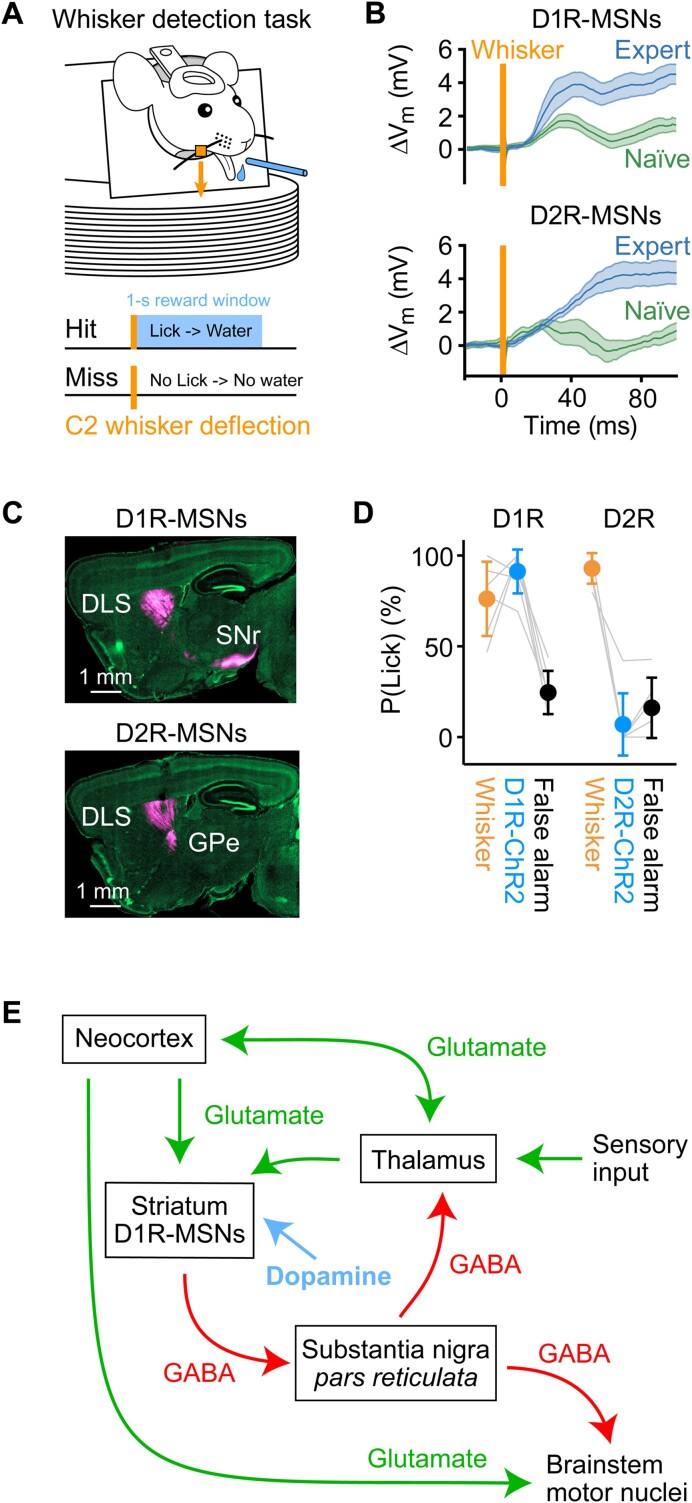
Striatal MSNs expressing D1Rs can drive goal-directed motor output and show enhanced fast sensory responses across learning. (A) Head-restrained thirsty mice can learn to lick a spout for a water reward in response to a whisker deflection (orange), which serves as a sensory cue predicting reward availability for 1 s with licking as the necessary goal-directed motor output to trigger reward delivery in Hit trials. (B) Whole-cell membrane potential (V_m_) recordings averaged across Hit trials for post hoc identified D1R-expressing and D2R-expressing MSNs in the DLS of expert (blue) or naïve (green) mice performing the whisker detection task. Whisker deflection evoked a larger depolarization in expert mice compared to naïve mice for both D1R-expressing and D2R-expressing MSNs. However, a fast (20-50 ms after whisker stimulus) sensory response appeared to increase specifically in D1R-expressing MSNs across learning.^85^ (C) Sagittal sections through mouse brains counterstained with 4′,6-diamidino-2-phenylindole, DAPI (green).^84^ An AAV was injected into the DLS in order to express fluorescent proteins to allow imaging of the cell bodies in the striatum and their axonal projections (magenta). Distinct classes of MSNs were defined by using two transgenic mouse lines in which Cre-recombinase was specifically expressed in either D1R- and D2R-expressing MSNs and injecting the DLS with a Cre-dependent AAV. Dopamine type 1 receptor-expressing MSNs strongly innervate the SNr, whereas D2R-expressing MSNs strongly innervate the GPe. (D) Channelrhodopsin-2 was expressed in either D1R- or D2R-expressing MSNs in the DLS of different mice, which were subsequently trained in the whisker detection task. ^84^ Once the mice were experts, whisker (orange) and catch (black) trials were randomly interleaved with trials containing a brief blue light pulse (blue) delivered to the DLS. Optogenetic stimulation of D1R-expressing MSNs evoked licking, but not optogenetic stimulation of D2R-expressing MSNs. Apparently, brief activation of D1R-expressing MSNs is sufficient to substitute for the whisker stimulation in this behavior. (E) A schematic circuit diagram that could account for some aspects of the learning and execution of goal-directed motor output in response to a sensory stimulus, as exemplified above by the transformation of a whisker deflection into goal-directed licking in the whisker detection task. Sensory input drives thalamic and cortical neurons, which in turn signal to the striatum. If the sensory input is paired with reward, then the sensory-evoked glutamatergic input from the thalamus and cortex will be accompanied by a dopaminergic reward signal, strengthening the excitation of D1R-expressing MSNs through LTP during reward-based learning. Enhanced sensory-evoked activity of D1R-expressing MSNs will inhibit neurons in SNr, in turn disinhibiting thalamus and brainstem motor nuclei, thus contributing to movement initiation such as licking for reward, causing further reinforcement of the sensorimotor transformation. Panel B is modified from,^85^ published under a Creative Commons License. Panels C and D are modified from,^84^ published under a Creative Commons License.

The hypothesis that dopamine signals might contribute to reward learning through modulating synaptic plasticity of specific neuronal circuits remains to be further tested in detail, but some experiments support the notion that striatal MSNs expressing D1Rs can contribute to driving goal-directed motor output and show enhanced fast sensory responses across learning, consistent with the dopamine hypothesis. Here, we will focus on a whisker detection task, which has been investigated in some detail with respect to D1R- and D2R-expressing MSNs across reward-based learning (Figure 4).^83–85^ In the whisker detection task, head-restrained thirsty mice learn to lick a spout in response to a brief single deflection of the C2 whisker in order to receive water reward (Figure 4A). Initially, mice are naïve to the reward-predicting rule, and they lick with equal probability independently of whisker deflection. Through trial-and-error learning across daily training sessions, mice receive reward, presumably at first by chance, by licking in the 1-s reward window that follows the 1-ms magnetic impulse applied to a metal particle attached to the C2 whisker serving as the tactile stimulus. After several training sessions, mice learn to lick reliably in response to whisker deflection, on each trial gathering a small droplet of water, accumulating rewards across the correct trials until sated. The hit rate (probability of licking in response to a whisker deflection) therefore increases across learning. Concomitantly, mice also learn to withhold licking at other times, presumably to reduce unrewarded effort. Thus, the false alarm rate (probability of licking in the absence of a whisker stimulus) decreases across learning.

Membrane potential (V_m_) recordings during the whisker detection task^84^, ^85^ were obtained from neurons located in the region of the DLS, known to receive direct glutamatergic input from the primary whisker somatosensory cortex (Figure 4B).^84^, ^86^, ^87^ Neurons were post hoc anatomically identified and colocalized with genetic markers to identify D1R- and D2R-expressing MSNs. Dopamine type 1 receptor-expressing MSNs in the DLS strongly innervate the SNr, whereas D2R-expressing MSNs in the DLS strongly innervate the GPe (Figure 4C).^84^ Averaged across hit trials for different recordings in different mice, both D1R- and D2R-expressing MSNs in expert mice showed an overall increased depolarization in response to whisker deflection compared to naïve mice (Figure 4B).^85^ In part, this is likely driven by enhanced glutamatergic input to the striatum from the cortex and thalamus during movements, including licking. The co-activation of D1R- and D2R-expressing MSNs is in good overall agreement with other recent studies.^88^, ^89^ Although more subtle, learning also appears to enhance a fast sensory response, specifically in D1R-expressing MSNs, occurring during a ∼20-50 ms period immediately after the whisker stimulus. One interesting hypothesis is that dopamine reward plasticity mediated via D1R signaling could contribute to potentiating glutamatergic whisker sensory input from the cortex or thalamus, thus giving rise to the observed fast sensory response in D1R-MSNs. Similarly, frequency-specific potentiation of corticostriatal synaptic transmission linked to reward-predicting tones as rats learned an auditory discrimination task has been reported.^90^

Optogenetic stimulation experiments were carried out to test for possible causal contributions of activity in D1R- and D2R-expressing neurons during execution of the whisker detection task (Figure 4D).^84^ A Cre-dependent virus was injected into the DLS of genetically engineered mice expressing Cre-recombinase in either D1R- or D2R-expressing MSNs. The mice were subsequently trained in the whisker detection task, and upon reaching high performance, trials with brief (50 ms) optogenetic stimuli were delivered through an optical fiber inserted into the DLS. The optogenetic stimulus trials were randomly interleaved with whisker stimulus trials and no-stimulus catch trials. Stimulation of D1R-expressing neurons evoked licking, but not the stimulation of D2R-expressing neurons. Brief activity in D1R-expressing neurons therefore seems to be sufficient for task execution with the optogenetic stimulus readily substituting for the whisker stimulus. The fast sensory-evoked depolarization of D1R-expressing neurons found in expert mice (Figure 4B) could therefore causally contribute to the learning and execution of the whisker detection task. These data are consistent with other studies indicating that optogenetic stimulation of D1R-expressing MSNs tends to invigorate and enhance movement production, whereas stimulation of D2R-expressing MSNs depresses movement initiation.^91–94^

In order to define hypotheses for further experimental testing, it might be useful to consider how different neuronal pathways might contribute to the transformation of a sensory input into a goal-directed motor output learned through dopamine reward signals (Figure 4E). Sensory information is signaled to the thalamus, which in turn innervates the cortex and striatum. Motor control is regulated by the neocortex, SNr, and other brain regions, which strongly innervate neuronal circuits in the brainstem and spinal cord, where motor neurons are located. Dopamine reward signals might serve to enhance sensory-evoked glutamatergic synaptic input to D1R-expressing MSNs through LTP. Enhanced D1R-expressing neuronal activity will release the inhibitory neurotransmitter GABA onto spontaneously active neurons in the SNr, thus reducing their firing rate. The SNr neurons are also inhibitory, and thus suppression of their firing has a disinhibitory effect upon downstream targets, such as the thalamus and brainstem motor nuclei.^93^, ^95^, ^96^ The net effect is increased motor drive, for example, enhanced probability of initiating a lick in the whisker detection task. The underlying mechanisms of reward learning might thus include a dopamine-dependent strengthening of feedforward synaptic neuronal circuits connecting a reward-predicting sensory stimulus with the execution of a motor command associated with reward delivery.

Many open questions remain before one could claim to have an understanding of the neuronal circuitry underlying the learning and execution of any specific goal-directed behavior. Even for the relatively simple whisker detection task discussed above, in which thirsty mice learn to lick a water reward spout in order to obtain a reward, many aspects remain unexplored. Many key causal tests of the specific hypothesis that dopamine-gated LTP of whisker sensory inputs to striatal D1R-expressing MSNs might underlie learning still need to be carried out. For example, direct manipulation of the dopamine signals has not yet been carried out during execution or learning of the whisker detection task, and neither have pharmacological manipulations targeting D1Rs or D2Rs. Furthermore, ideally, the same neurons and synaptic inputs would be studied longitudinally across learning to investigate in further detail the underlying mechanisms and sites of synaptic plasticity, as well as the patterns of neuronal activity that induce the plasticity.

From a higher-level perspective, we also need to consider how water becomes rewarding when mice are thirsty, how this motivates mice to lick, and how receiving a water reward (or a sensory cue predicting upcoming water reward, such as the whisker stimulus in the whisker detection task) generates a dopamine signal. Some aspects of how thirst is represented in the brain are beginning to be understood, but it remains difficult to assemble an integrative view of how this impacts behavior. Blood osmolality is first sensed by neurons in the subfornical organ and the organum vasculosum of the lamina terminalis, structures that lack the blood-brain barrier. Interestingly, optical stimulation of a genetically defined subset of neurons in the subfornical organ (expressing CaMKII, nitric oxide synthase, and ETV-1) immediately triggers drinking behavior, and these neurons are also activated by thirst.^97^ Such optogenetic stimulation of the subfornical organ has been shown to be negatively reinforcing and thus possibly generating an aversive state that motivates mice to find and consume water. Neurons in the subfornical organ in turn project to various other brain regions, including hypothalamic regions such as the median preoptic nucleus, supraoptic nucleus, and the paraventricular hypothalamic nucleus, and indeed, optogenetic stimulation of neurons in the median preoptic nucleus also drives water-seeking behavior.^98,99^ Perhaps via hypothalamic neurons, the thirst state can change cortical sensory processing,^100^ and optogenetic manipulation of thirst neurons has been shown to give rise to highly distributed changes in neuronal activity patterns and sensorimotor processing during goal-directed behavior motivated by thirst,^101^ but the causal mechanisms linking changes in diverse classes of neurons in different brain regions remain to be determined. Interestingly, neurons in the lateral hypothalamus appear to signal thirst and fluid balance states to dopamine neurons in the VTA contributing importantly to the learning of which foods and fluids are rehydrating.^102^

In conclusion, remarkable progress has been made linking striatal dopamine signals to reward learning, but much remains to be learned.

## Data Availability

No new data were generated or analyzed in support of this research.
